# Integrating Deep Learning Models with Genome-Wide Association Study-Based Identification Enhanced Phenotype Predictions in Group A *Streptococcus*

**DOI:** 10.4014/jmb.2411.11010

**Published:** 2025-03-26

**Authors:** Peng-Ying Wang, Zhi-Song Chen, Xiaoguo Jiao, Yun-Juan Bao

**Affiliations:** School of Life Sciences, Hubei University, Wuhan 430062, P.R. China

**Keywords:** Group A *Streptococcus*, deep learning, phenotype prediction, genome-wide association study

## Abstract

Group A *Streptococcus* (GAS) is a major pathogen with diverse clinical outcomes linked to its genetic variability, making accurate phenotype prediction essential. While previous studies have identified many GAS-associated genetic factors, translating these findings into predictive models remains challenging due to data complexity. The current study aimed to integrate deep learning models with genome-wide association study-derived genetic variants to predict pathogenic phenotypes in GAS. We evaluated the performance of several deep neural network models, including CNN, ResNet18, LSTM, and their ensemble approach in predicting GAS phenotypes. It was found that the ensemble model consistently achieved the highest prediction accuracy across phenotypes. Models trained on the full 4722-genotype set outperformed those trained on a reduced 175-genotype set, underscoring the importance of comprehensive variant data in capturing complex genotype-phenotype interactions. Performance changes in the reduced 175-genotype set compared to the full-set genotype scenarios revealed the impact of data dimensionality on model effectiveness, with CNN remaining robust, while ResNet18 and LSTM underperformed. Our findings emphasized the potential of deep learning in phenotype prediction and the critical role of data-model compatibility.

## Introduction

Group A *Streptococcus* (GAS) is a significant human pathogen responsible for a broad spectrum of diseases, ranging from superficial infections such as pharyngitis and impetigo to severe invasive diseases like necrotizing fasciitis and streptococcal toxic shock syndrome [1, 2]. The ability of GAS to cause diverse clinical manifestations is influenced by its genetic variability, making the identification of genotype-phenotype relationships critical for understanding its pathogenicity and developing effective therapeutic strategies.

Advances in genotype-phenotype relationship studies have significantly enhanced our understanding of the genetic basis of complex traits in pathogens like GAS. Previous researches have identified numerous genetic factors, such as single nucleotide polymorphisms (SNPs), insertions/deletions (InDels), and gene absence/presence which are associated with virulence, antibiotic resistance, or pathogenicity in GAS [3-5]. For instance, the deletion of the gene cluster encoding the bacterial envelope (*hasABC*) and the overexpression of the cytotoxic gene cluster (*nga-slo*) are significantly related to the serious skin infection caused by the M serotype-89 strains [6, 7]. One non-synonymous mutation in nicotine adenine dinucleotide glycohydrolase (*nga*) and two mutations in its promoter region are significantly related to the M serotype-1 strains causing pharyngitis [8]. The introduction of supervirulence factors (*ssa, speC, spd1*) and tetracycline and macrolide resistance genes (*tetM, ermB*) due to the insertion of two large genomic fragments are the main reasons that M serotype-12 strains are resistant to drugs and caused scarlet fever [9, 10]. These virulence genes play pivotal roles in many aspects of GAS pathogenesis, such as enhanced colonization, transmission, or immune evasion. The impact of supervirulence genes can extend beyond individual infections to cause population-level epidemiology with the notable examples of Sda1-encoding M serotype-1 M1T1 clones [2] and Spd1/SpeC-encoding M serotype-12 isolate HKU16 [11, 12].

Recently, several studies employed genome-wide association study (GWAS) to GAS genomes of specific serotypes [13] or specific phenotypes [14, 15] to identify hundreds of genetic factors associated with GAS pathogenesis. Despite the significant progresses made through GWAS, translating these findings into predictive models that can effectively predict phenotypic outcomes remains a substantial challenge. Current studies of predicting phenotypes from genetic data mainly utilized the approaches of traditional machine learning methods, such as logistic regression, support vector machines, random forest, and gradient boosting [16, 17]. While these methods are effective in specific contexts, they failed to capture non-linear interactions within genetic data due to their mathematical assumption of linearity and independence between genetic features. Furthermore, these methods also face challenges in scalability and robustness when handling high-dimensional data [18, 19]. Advances in artificial intelligence (AI), particularly deep learning, have opened new avenues for modeling complex biological data. Deep learning models, such as Convolutional Neural Networks (CNNs), Recurrent Neural Networks (RNNs), Long Short-Term Memory (LSTM), and their variants, have demonstrated remarkable success in various biological applications [20-22], such as disease susceptibility prediction [23, 24], gene expression estimation [25, 26], protein-DNA interaction modeling [27, 28], natural selection detection [29-31], and recombination rate estimation [32-34]. These models excel at capturing non-linear relationships in high-dimensional datasets, making them ideal for genotype-phenotype associations in complex genetic settings like GAS. Recently, the application of deep learning models in predicting phenotypes is just emerging, with significant potential to enhance phenotype prediction accuracy [35, 36]. DeepManReg improves phenotype prediction by aligning multi-modal features onto a shared latent space to capture cross-modal interactions, achieving higher accuracy and AUC than three baseline models in both an image dataset and a single-cell multi-modal dataset [35]. DeepGS effectively prioritizes genotype-phenotype associations and feature importance in high-dimensional genomic data of 33,709 markers, outperforming five baseline models in terms of PCC (Pearson's correlation coefficient) and MNV (Mean normalized discounted cumulative gain value) for predicting eight wheat traits [36].

A key challenge in predicting phenotypes from genetic data is managing the high dimensionality and sparsity of the data, as well as the heterogeneity of genetic variants. Traditional approaches of genotype-phenotype association studies usually focused on specific types of variants, like SNPs, without accounting for broader genomic context. Our current study aims to address this limitation by combining deep learning models with comprehensive genetic data derived from a novel GWAS approach, *i.e.*, compGWAS, previously developed by us [37]. This new approach is able to identify functional unit-level variants of protein-coding regions (CDS) and non-coding regions (nonCDS) induced from SNPs and InDels, thus providing a more complete dataset for phenotype prediction.

We transformed the functional unit-level variants to categorical genotypes to train deep learning models like CNNs, ResNet18, and LSTM networks. This combined approach allows the prediction models to uncover complex interactions among the genetic variants, improving the accuracy of phenotype predictions.

Our approach in this study has several advantages. First, we leveraged deep learning models to handle high-dimensional and sparse genetic data more effectively than traditional methods. The deep learning models excel at capturing the complex, non-linear relationships in genomic data that are often ignored by conventional genotype-phenotype association approaches, like GWAS. Second, we implemented an ensemble method that integrates predictions from different model architectures, such as CNNs, ResNet18, and LSTM networks. This ensemble approach was shown to be able to improve both accuracy and robustness by taking the advantages of individual models.

By integrating comprehensive genetic information with powerful AI models, we offered a computational framework that applies deep learning models for predicting disease phenotypes based on GWAS-derived genetic variants, which was shown to not only enhance phenotype prediction, but also deepen our understanding of the genetic underpinnings of GAS pathogenicity.

## Materials and Methods

### Genomic Data Preparation and Preprocessing

We collected genome sequencing data and annotation information for 2602 strains of GAS from databases, such as the NCBI Genbank database (ftp://ftp.ncbi.nlm.nih.gov/genomes/genbank) and EBI (https://www.ebi.ac.uk/) (as of Feb. 2021). Through literature research and database searches in NCBI (https://www.ncbi.nlm.nih.gov/data-hub/genome/?taxon=1314), ATCC (www.atcc.org), and NCTC (https://www.culturecollections.org.uk/collections/nctc.aspx) (as of Aug. 2021), we extracted multiple types of clinical information, including pathogenic phenotypes, serotypes, and infection sites. The strains isolated from population-level experiments were excluded from the analysis since those strains may produce amplified signals induced from special events and obscure true phenotype-genotype associations. The strains were further selected from the top four phenotypes, *i.e.*, Invasive (INV), Superficial Soft Tissue Infection (SSTI), Pharyngitis (PHY), and Deep Soft Tissue Infection (DSTI), which have sufficient sample sizes (>150) for model training and testing. Ultimately, a total of 1349 strains were used for downstream analysis. A large amount of clinical information of GAS strains were derived from the study by Davies and *et al*. [15].

### Genotype-Phenotype Data Processing

In our previous study [37], we conducted a GWAS analysis using the logistic regression model and identified 4722 genotypes significantly associated with the four phenotypic traits (INV, PHY, SSTI, DSTI), including 4360 SNP-based genotypes, 101 CDS-based genotypes, and 261 nonCDS-based genotypes (Supplementary S1 and Supplementary S2). The CDS-based genotypes were defined as intact or inactivated, indicating the coding status of CDS, which could be intact or inactivated by mutations (SNPs or InDels). The nonCDS-based genotypes were defined as intact or mutated, indicating the mutational status of a nonCDS region. In a window of a nonCDS region, if any InDel minor allele was identified in one or more genomic loci of a strain, the mutational status of this window was considered to be mutated, and intact otherwise. A fixed window size of 200 bp was used to walk at a step of 20 bp along the genome. The genotypes of all windows in a single nonCDS region were recorded. These genotypes were screened through a multi-step process to refine association significance. For SNP-based variants, they were first selected based on the association *p*-value significance from pair-wise phenotype-genotype GWAS analysis using the maximal curvature method [38]. The final *p*-value thresholds were determined for each phenotype pair as following: *p* < 0.0251 for INV-PHY, *p* < 0.00316 for INV-SSTI, *p* < 0.0126 for INV-PHYSSTI, *p* < 0.0251 for INV-RF, *p* < 0.0355 for PHY-RF, *p* < 0.01 for PHY-SSTI, and *p* < 0.00447 for RF-SSTI. Then, linkage-disequilibrium (LD) pruning and functional annotation were used to reduce redundancy from correlated SNPs. Only SNPs with low LD (pair-wise correlation r^2^ <0.8) and non-synonymous functional annotation were selected. For CDS-based and nonCDS-based variants, the association *p*-value thresholds were determined manually due to the low number of variants in these two categories. The *p*-value thresholds for CDS-based variants were *p* < 0.05 for all phenotype pairs, and those for nonCDS-based variants were as following: *p* < 0.05 for INV-PHY, *p* < 0.015 for INV-SSTI, *p* < 0.03 for INV-PHYSSTI, *p* < 0.05 for INV-RF, *p* < 0.05 for PHY-RF, *p* < 0.03 for PHY-SSTI, and *p* < 0.04 for RF-SSTI. The CDS-based variants were selected only based on the *p*-value. For nonCDS regions containing multiple 200-bp windows satisfying the *p*-value threshold, the windows with the lowest *p*-values were selected for each region. Each genotype was transformed to binary data (0 or 1, where 0 represents the major allele and 1 represents the minor allele) for the 1349 GAS strains, resulting in a genotype dataset. This dataset was divided into training and testing sets at a ratio of 4:1, with 1066 training samples (436 INV, 321 SSTI, 174 DSTI, 135 PHY) and 283 testing samples (122 INV, 77 SSTI, 35 DSTI, 49 PHY).

### Model Construction

We constructed three types of neural network models, *i.e.*, CNN, ResNet18 [39], and LSTM [40]. For each model, we optimized the hyperparameters by performing 5-fold cross-validation on the training dataset. The hyperparameters yielding the best average prediction accuracy across the 5 folds were selected for further analysis of each model.

The CNN model architecture comprised an input layer with 4563 input nodes (after filtering out 159 SNP genotypes to meet the input dimension requirements of the CNN model), three convolutional layers, two fully connected layers, and one output layer (Supplementary S1). Each convolutional layer is followed by a batch normalization (BN) layer and a ReLU activation function. Pooling and Dropout layers were used to reduce overfitting and improve generalization of the model. The detailed architecture of the model is presented in Table 1.

Hyperparameters for optimization included weight decay, dropout rates, weight initialization, optimizer, learning rate, and batch size, with 2000 combinations tested. The final optimized hyperparameters were: weight decay = 10^-8^, no dropout, weight initialization enabled, Adam optimizer with learning rate = 0.00012, and batch size = 64. The number of epochs was determined to be 100 based on the learning curve of the cross-validation loss.

We adapted the ResNet18 model without pre-trained weights to our dataset by modifying the input layer to accommodate the 4563 genotypes (after filtering out 159 SNP genotypes to meet the input dimension requirements of the ResNet18 model) and the output layer to classify the four phenotypes (Supplementary S1). It includes a series of convolutional layers, BN layers, ReLU activation functions, and pooling layers. The detailed architecture of the ResNet18 model, including the output shapes at each layer, is presented in Table 2.

Each BasicBlock in the ResNet18 architecture included two convolutional layers, each followed by a BN layer and a ReLU activation function (Table 3). Additionally, there is a residual connection that adds the input of the block to its output. This architecture helps addressing the vanishing gradient problem for deep neural networks.

Hyperparameters for optimization included weight decay, optimizer, learning rate, and batch size, with 1000 combinations tested. The optimized hyperparameters were: weight decay = 10^-4^, SGD optimizer with learning rate = 0.0092, and batch size = 64. The number of epochs was determined to be 182 based on the learning curve of the cross-validation loss.

The LSTM model architecture included an input layer with 4700 input nodes (after filtering out 22 SNP genotype loci to meet the input dimension requirements of the LSTM model), one LSTM layer with a specified number of hidden units, and a fully connected layer (classifier) with dropout regularization applied (Table 4 and Supplementary File S2). The input data was processed through the LSTM layer, which captures temporal dependencies, and then the output was passed through a dropout layer and a fully connected layer to produce the final class predictions.

Hyperparameters for optimization included weight decay, number of hidden units, number of LSTM layers, dropout rates, optimizer, learning rate, and batch size, with 1000 combinations tested. The optimized hyperparameters were: no weight decay, 512 hidden units, 2 LSTM layers, dropout rate = 0.1, Adam optimizer with learning rate = 0.0042, and batch size = 64. The number of epochs was determined to be 164 based on the learning curve of the cross-validation loss.

### Model Training and Evaluation

**Fig. 1:**
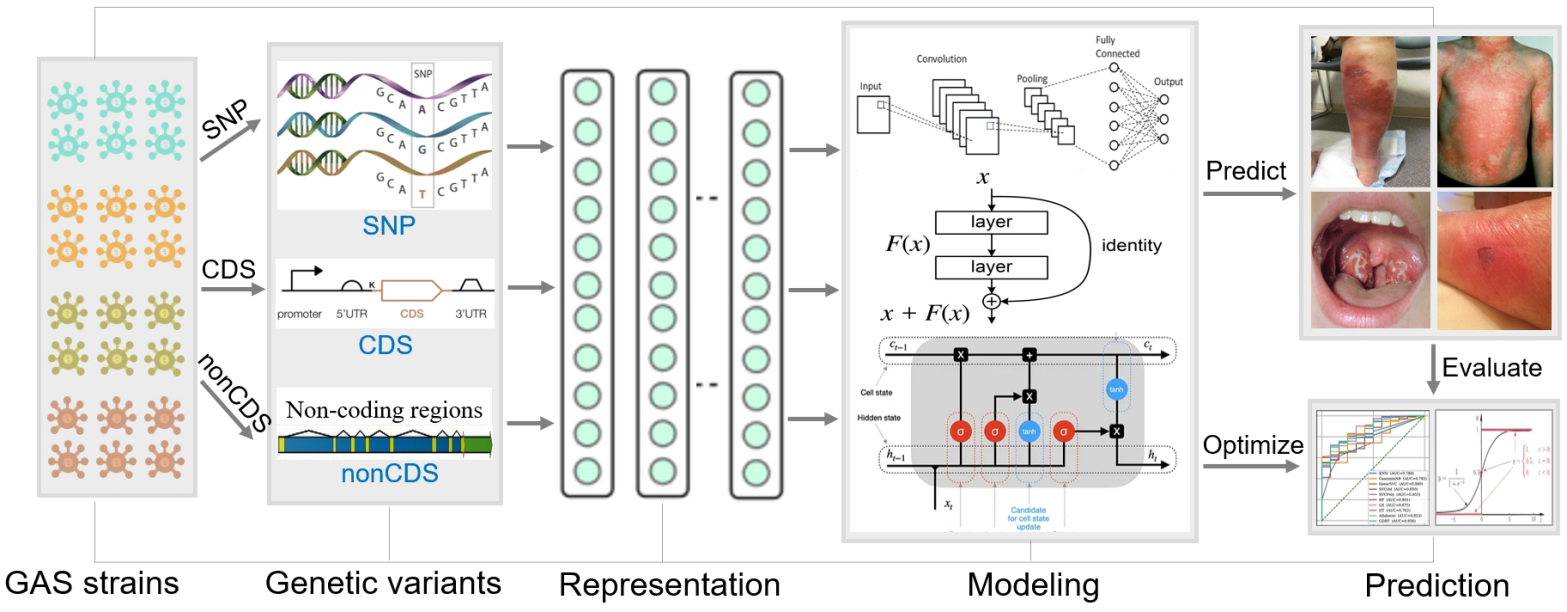
The overview of the workflow. This figure provides an overview of the entire analysis workflow, beginning with the integration of multiple genotype data, followed by genotype-phenotype data preparation, and progressing to the construction, training, and evaluation of the neural network models.

Given the smaller sample size of the PHY phenotype, we performed 3x upsampling to balance the sample sizes of distinct phenotypes in the training dataset. Each model was trained on the training dataset with the best hyperparameters identified through 5-fold cross-validation. The trained models were evaluated on the testing dataset using multiple performance metrics: accuracy, sensitivity, specificity, precision, F1 score, micro AUC, and macro AUC. To improve the prediction accuracy, we implemented an ensemble method to combine the predictions from the three individual models. For each testing sample, the ensemble prediction was determined based on the model of the highest accuracy for that sample. The workflow in Fig. 1 provides an illustration of our approach for integrating genotype-phenotype data with neural network models to predict pathogenic phenotypes of GAS strains.

## Results

### Correlation Analysis of Training Samples

**Fig. 2:**
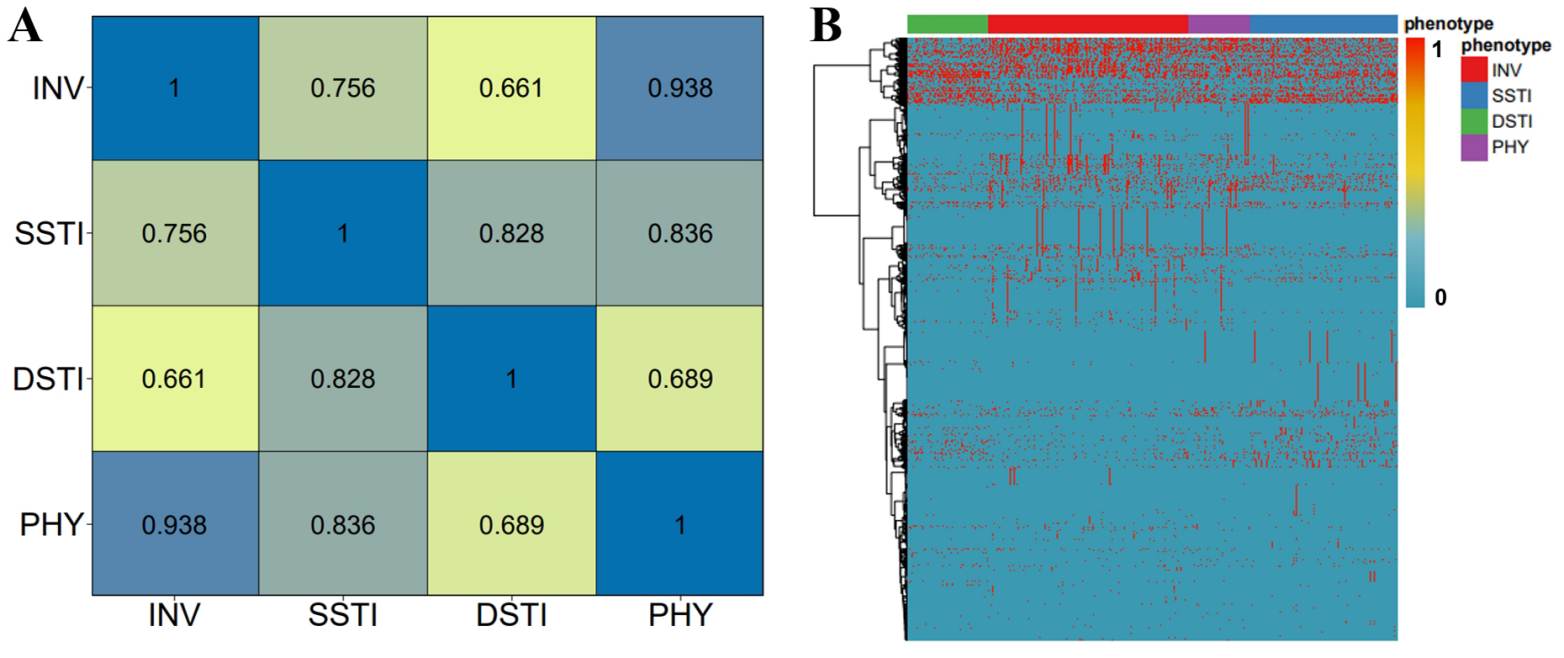
The correlation matrix between the four studied phenotypes and heatmap representation of the genotype matrix for the training set samples. (**A**) The Pearson correlation between the four phenotypes, *i.e.*, INV, SSTI, DSTI, and PHY. (**B**) The heatmap provides a visual representation of the genotypes of the 4722 genomic variants across the training samples for the four phenotypes. The genotypes are indicated with “1” and “0” (red and blue in the colored bar). The heatmap shows the presence (red, 1) or absence (blue, 0) of the genomic variants. The dendrogram on the left shows the clustering of the genomic variants.

We first performed a Pearson correlation analysis for the training samples using the minor allele frequency (MAF) of the 4722 genotypes. The analysis focused on the correlation coefficients between the four phenotypes, *i.e.*, INV, SSTI, DSTI, and PHY and produced a correlation matrix (Fig. 2A). The matrix illustrates the pairwise correlation between the four phenotypes in the training dataset. The values of coefficients ranged from 0.661 to 0.938, indicating varying degrees of correlation. Notably, the highest correlation was observed between INV and PHY (0.938), suggesting a strong genetic similarity between these two phenotypes. SSTI also showed a high correlation with both PHY (0.836) and DSTI (0.828), indicating shared genetic determinants. These findings are crucial for understanding the genetic relationships across phenotypes and developing accurate predictive models.

The heatmap representation of the genotype matrix reveals distinct patterns across phenotypes, highlighting the suitability of the data for developing discriminative models (Fig. 2B). Certain variants are predominantly associated with specific phenotypes, which allows for developing effective prediction models.

### Performance of Deep Learning Models in Predicting the Four Phenotypes

**Fig. 3:**
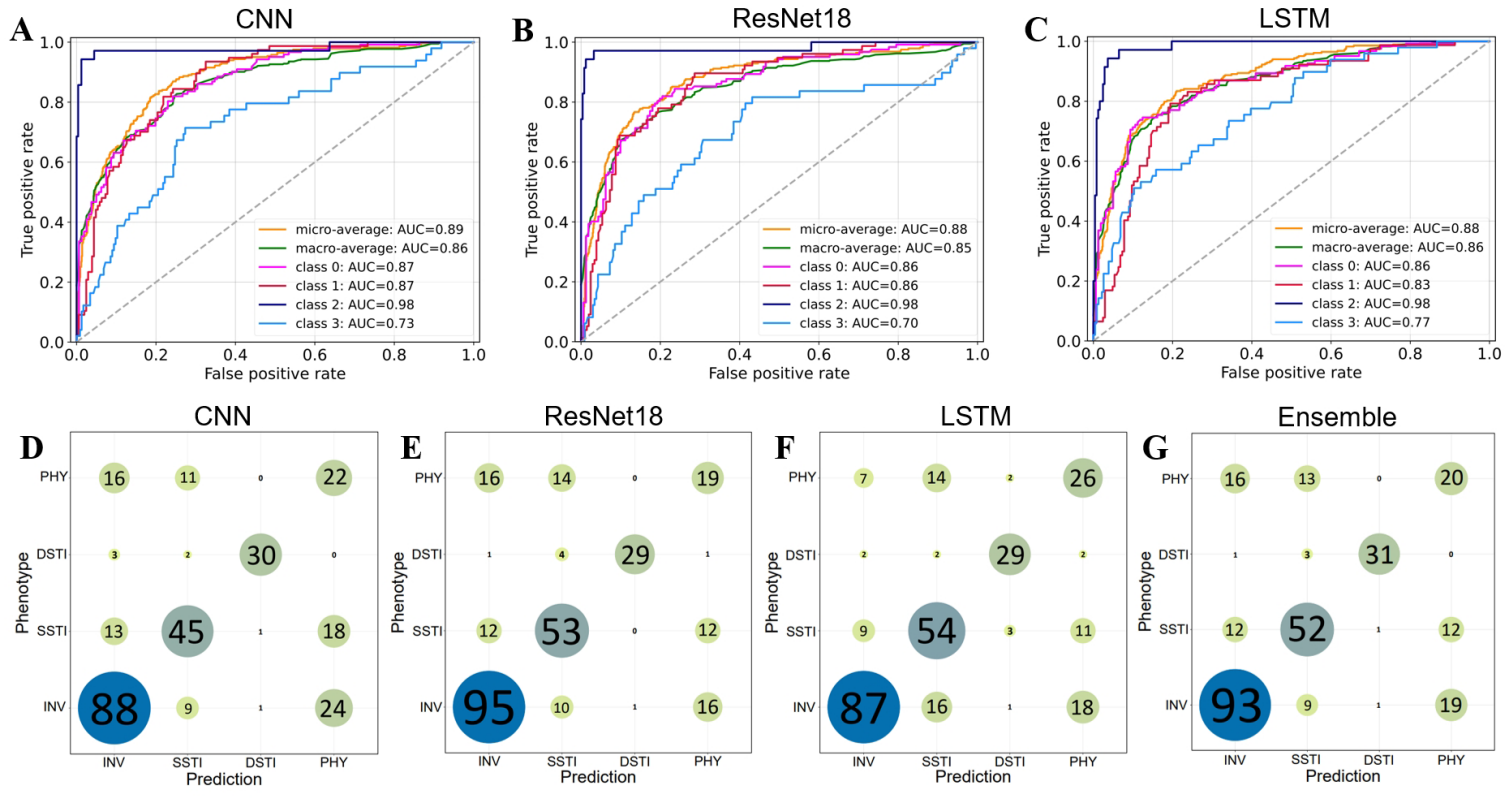
The ROC curve and confusion matrix of predicting models for the four phenotypes in the testing dataset. (**A-C**) the ROC curve of the deep learning models CNN (**A**) ResNet18 (**B**) and LSTM (**C**). Class 0: INV, class 1: SSTI, class 2: DSTI, and class 3: PHY. (**D-G**) The confusion matrix of the deep learning models CNN (**D**) ResNet18 (**E**) and LSTM (**F**), as well as their ensemble analysis (**G**).

The performance of the three neural networks (CNN, ResNet18, and LSTM) and their ensemble model was evaluated for phenotype prediction using a training dataset of 1066 samples and a testing dataset of 283 samples (see Materials & Methods). Overall, the deep learning models showed strong predictive performance, with the ensemble model delivering the best results across most metrics (Table 5). The ensemble achieved a Top-1 Accuracy of 0.693, matching the performance of ResNet18 and LSTM. The Sensitivity, Specificity, Precision, and F1 Score of the ensemble model were improved by 1.59%, 0.38%, 1.12%, and 1.63%, respectively than the average value of the three base models, demonstrating the advantage of combining multiple models for more consistent predictions. ROC curves and AUC scores further confirmed strong performance across most phenotypes, particularly for DSTI, where AUC values reached around 0.98 (Fig. 3A-3C). The slight variations between the individual models highlighted their respective strengths in different metrics, while the ensemble approach successfully leveraged these strengths, leading to the highest overall prediction accuracy and robustness across all phenotypes.

When examining the model performance for each individual phenotype, we found that all models show lower performance in the metrics for predicting PHY than other phenotypes (Fig. 3A-3C and Table S1). It was corroborated by the high proportion of misclassifications of PHY with INV or SSTI as shown in the confusion matrices (Fig. 3D-3G). The misclassification is probably related with the high genetic correlation between PHY and INV/SSTI, which negatively influences the overall and phenotype-specific performance of the models (Fig. 2A). It was noted that the prediction accuracy was significantly improved by 30.74% when considering the top two prediction classes (Top-2 Accuracy in Table 5). These results suggest that the complex relationship between PHY and other phenotypes poses a key challenge for accurate phenotype prediction.

### Performance of Deep Learning Models in Predicting Three Phenotypes

**Fig. 4:**
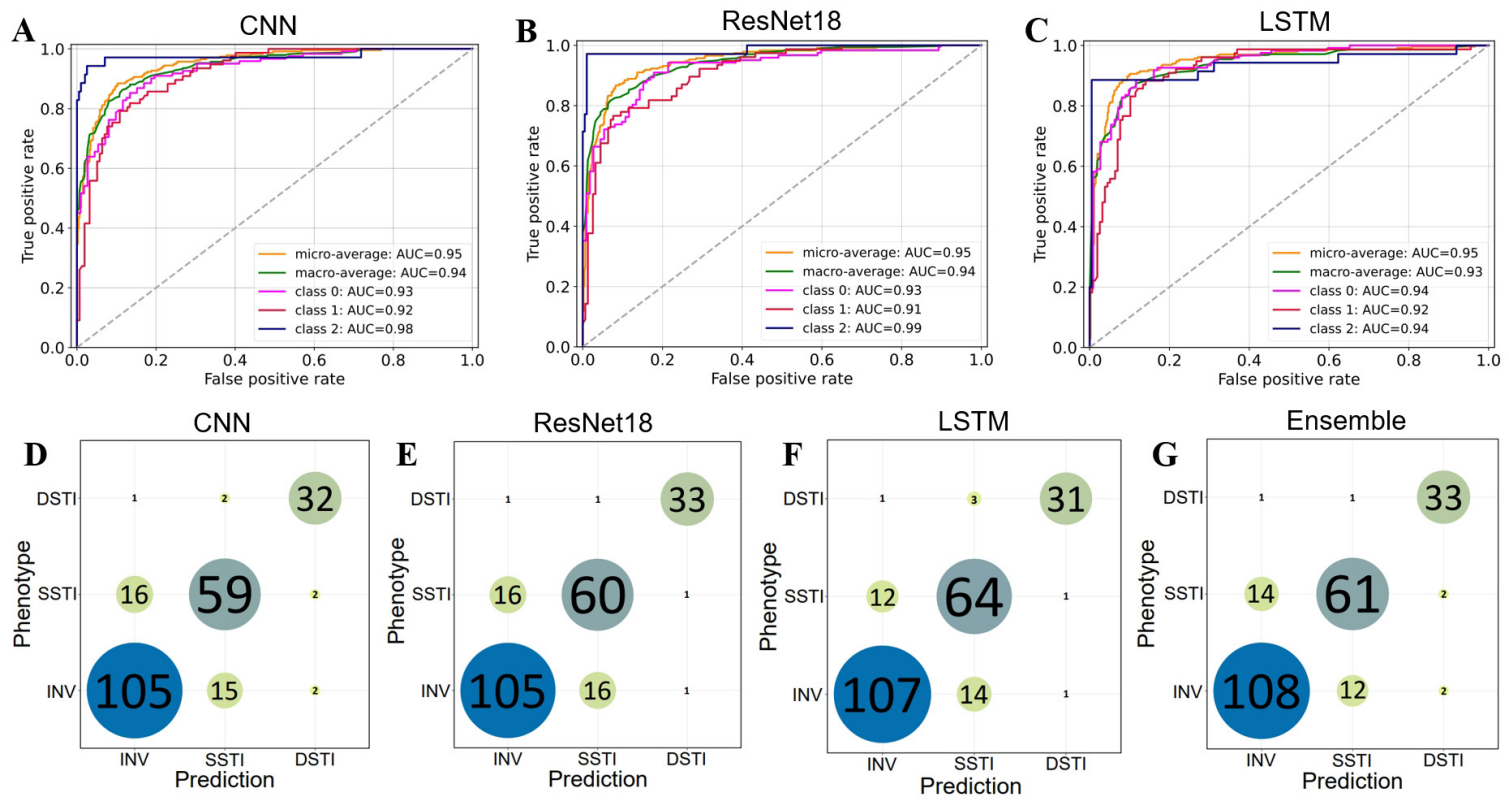
The ROC curve and confusion matrix of predicting models for the three phenotypes in the testing dataset. (**A-C**) the ROC curve of the deep learning models CNN (**A**), ResNet18 (**B**), and LSTM (**C**). Class 0: INV, class 1: SSTI, and class 2: DSTI. (**D-G**) The confusion matrix of the deep learning models CNN (**D**), ResNet18 (**E**), and LSTM (**F**), as well as their ensemble analysis (**G**).

Considering the high similarity and strong confusion of the phenotype PHY with the other two phenotypes (Figs. 2A and 3D-3G), *i.e.*, INV and SSTI, we suppose that excluding PHY might improve the prediction performance of the models. Therefore, we performed model training and evaluation only for the remaining three phenotypes, *i.e.*, INV, SSTI, and DSTI (Supplementary S3 and S4). Compared to the four-phenotype predictions, the three-phenotype models showed notable improvements in performance metrics and reduction in confusion (Table 6 and Fig. 4). Specifically, Top-1 Accuracy increased by an average of 24.8%, Sensitivity by 27.6%, Precision by 25.4%, F1 Score by 26.8%, Micro AUC by 7.5%, and Macro AUC by 9.3% (Table 6 and Fig. 4A-4C). The increased performance in the metrics is also supported by the lower misclassification and decreased confusion between the three phenotypes (Fig. 4D-4G). The increase in F1 scores across all models, particularly in the ensemble (from 0.687 to 0.869), reflects the improved balance between precision and specificity (Table 6). These results highlight that the exclusion of confusing samples significantly enhanced model accuracy and robustness in distinguishing between phenotypes.

Among the four models, the ensemble model continued to provide the most balanced and superior performance across all metrics. It achieved a Top-1 accuracy of 0.863 and a Top-2 accuracy of 0.987, the highest among all models. The Sensitivity, Specificity, Precision, and F1 Score of the ensemble model were improved by 1.79%, 0.88%, 0.7%, and 1.28% respectively than the average value of the three base models. With Sensitivity at 0.873, Specificity at 0.921, Precision at 0.865, and F1 Score at 0.869, the ensemble approach leveraged individual model strengths for consistent improvement across phenotypes (Table S2).

In summary, removing the PHY phenotype resulted in significant enhancement in the prediction performance of deep learning models across all metrics. Particularly, the ensemble approach demonstrated the highest overall accuracy and reliability, supporting the effectiveness of combining multiple models for phenotype prediction.

### Comparison of Model Performance with Full and Reduced Genotypic Sets

In our previous GWAS analysis [37], we obtained a non-redundant biomarker set consisting of 175 non-collinear genotypes (175-genotype set) by applying the Least Absolute Shrinkage and Selection Operator (LASSO) regression on the full set of 4722 genotypes (full-set) to remove collinearity among genotypes (Supplementary File S5). We then compared the performance of deep learning models trained on the full-set genotypes versus deep learning models trained on the 175-genotype set, as well as the traditional logistic regression model trained on the 175-genotype set (Table 7).

Using the full-set genotypes, deep learning models (CNN, ResNet18, and LSTM) demonstrated notably higher prediction performance compared to models trained on the reduced 175-genotype set. On average, metrics improved by 6.21% for Top-1 Accuracy, 4.97% for Precision, and 5.45% for F1 Score. Compared to logistic regression models trained on the 175-genotype set, deep learning models trained on the full-set also demonstrated superior performance across all metrics, with an average improvement of 5.07% in Top-1 Accuracy, 3.37% in Precision, and 4.76% in F1 Score. The superior performance of the deep learning models on the full-set genotypes highlights the capability of deep learning models in capturing complex feature patterns from large-scale data.

For models trained on the reduced non-redundant 175-genotype set, CNN outperformed the logistic regression model, while ResNet18 and LSTM underperformed. This discrepancy may stem from the structural and functional differences among these models. CNNs were designed to focus on spatially local dependencies between data features through convolutional layers, which can effectively capture essential feature patterns even with a small input size. This spatial sensitivity enables CNNs to perform reliably with a low number of features without overfitting, since the low number of parameters and shared weights reduce model complexity, which are especially fit for low-dimensional datasets [41, 42].

Conversely, ResNet18’s deep architecture with residual connections was structured to capture complex hierarchical patterns across large input spaces [39], making it less suited for the reduced 175-genotype set. Additionally, when feature input is minimal, the complex architecture of ResNet18 may become overparameterized, leading to overfitting and reduction in predictive accuracy. Studies suggested that residual networks, due to their reliance on hierarchical feature extraction, generally achieve optimal performance with richer feature input [43, 44].

LSTM, optimized for sequence data, was inherently designed to capture long-term dependencies and relationships across sequential data points. Without an inherent temporal or sequential structure for the 175-genotype set, the LSTM is unable to utilize its architecture effectively, limiting its ability to detect non-sequential patterns among the reduced feature set. This outcome aligns with previous findings that LSTMs often underperformed on non-sequential, low-dimensional tasks [45, 46]. Furthermore, LSTMs often contained a large number of parameters to capture dependencies across time steps, which can lead to overparameterization and lower performance with a reduced feature set.

These results highlight two key conclusions. First, deep learning models trained on the full set of 4722 genotypes outperform models trained on a reduced set of 175 genotypes, as well as traditional logistic regression models, underscoring the ability of deep learning models to capture complex patterns or inner interactions among a large number of genetic variants. Secondly, while CNN remains robust even with a smaller number of features, more complex models like ResNet18 and LSTM may require larger feature sets to fully capture variant relationships and perform effectively. This differential performance highlights the importance of aligning model architecture with data dimensionality in genomic prediction tasks.

## Discussion

Our study demonstrated the potential of deep learning models in predicting pathogenic phenotypes by leveraging genetic variants identified through GWAS. The ability of these models to capture complex, non-linear relationships among genetic variants sets them apart from traditional machine learning models like logistic regression, which is unable to process interactions and dependencies between large numbers of variants. Specifically, our results emphasized that integrating these advanced deep learning models with comprehensive genetic data significantly enhances prediction accuracy and robustness.

One of the key findings in this study is the superior performance of the ensemble model, which combined the strengths of CNN, ResNet18, and LSTM architectures. The ensemble model demonstrated consistently higher accuracy across phenotypes, including DSTI and INV, surpassing the performance of individual models. This improved performance likely stemmed from the ensemble approach's ability to mitigate the weaknesses of individual architecture, such as CNN's limited sequential modeling capability or LSTM's higher complexity.

Our findings underscored the inherent complexity of genetic interactions in predicting pathogenicity, a challenge that traditional methods, such as logistic regression often failed to address. Traditional models may rely heavily on assumptions of linearity and independence, which limits their ability to account for the interactions between genetic variants. In contrast, deep learning models excel at capturing these variant interactions. For example, when using the full set of 4722 genotypes, the CNN, ResNet18, and LSTM models significantly outperformed traditional logistic regression models trained on a reduced biomarker set. This suggests that deep learning models, with their capacity for handling large and complex datasets, are better suited for these types of genomic analyses.

However, our study also brings attention to some critical challenges that need to be addressed in future work. A major limitation is the relatively small sample size and the lack of global diversity in our dataset. Larger and more diverse datasets representing various clinical and geographic settings would be helpful to improve model generalization.

Moreover, while deep learning models exhibited impressive predictive performance, their biological interpretability remains a key challenge. The “black-box” nature [47, 48] of these models makes it difficult to derive biologically meaningful insights from their predictions. Enhancing interpretability through methods such as saliency maps [49, 50], feature importance [51], or integrating domain knowledge [52, 53] into the model design could provide more actionable insights into the genetic underpinnings of pathogenicity.

Future research should also explore the integration of additional omics data [54-56], such as transcriptomics, proteomics, and metabolomics, to further refine phenotype predictions. Combining multiple layers of biological information could provide a more holistic view of the molecular mechanisms driving GAS pathogenicity, and potentially improve the accuracy and applicability of predictive models.

In conclusion, our study demonstrated the feasibility and effectiveness of applying deep learning models to predict pathogenic phenotypes from genetic variants carefully selected by traditional GWAS methods. By leveraging GWAS results and advanced deep learning models, our findings provided a paradigm for future research aiming at improving phenotype prediction or disease diagnosis.

## Supplemental Materials

Supplementary data for this paper are available on-line only at http://jmb.or.kr.



## Figures and Tables

**Table 1:** Network architecture of the CNN model.

Layer type	Description	Output shape
Input	4563 genotypes	(39, 39, 3)
Convolution, BN, ReLU	3×3 filter, 64 output channels, stride 1, padding 1	(39, 39, 64)
Max Pooling, Dropout	2×2 kernel, stride 2, padding 0	(19, 19, 64)
Convolution, BN, ReLU	3×3 filter, 128 output channels, stride 1, padding 1	(19, 19, 128)
Max Pooling, Dropout	2×2 kernel, stride 2, padding 0	(9, 9, 128)
Convolution, BN, ReLU	3×3 filter, 256 output channels, stride 1, padding 1	(9, 9, 256)
Max Pooling, Dropout	4×4 kernel, stride 4, padding 0	(2, 2, 256)
Flatten	Flatten the tensor	(1024, 1)
Fully Connected, ReLU		(256, 1)
Fully Connected, ReLU		(256, 1)
Fully Connected Output		(4, 1)

The output shape is given as (width, height, channels). BN denotes batch normalization.

**Table 2:** Network architecture of the ResNet18 model.

Layer type	Description	Output shape
Input	4563 genotypes	(39, 39, 3)
Convolution, BN, ReLU	3×3 filter, 64 output channels, stride 1, padding 1	(39, 39, 64)
Max Pooling	3×3 kernel, stride 2, padding 1	(20, 20, 64)
conv2_x	2 BasicBlocks (3×3 filter, 64 output channels)	(20, 20, 64)
conv3_x	2 BasicBlocks (3×3 filter, 128 output channels, stride 2)	(10, 10, 128)
conv4_x	2 BasicBlocks (3×3 filter, 256 output channels, stride 2)	(5, 5, 256)
conv5_x	2 BasicBlocks (3×3 filter, 512 output channels, stride 2)	(3, 3, 512)
Adaptive Avg Pooling	Output size (1, 1)	(1, 1, 512)
Flatten	Flatten the tensor	(512, 1)
Fully Connected Output		(4, 1)

The output shape is given as (width, height, channels). BN denotes batch normalization.

**Table 3:** BasicBlock architecture in ResNet-18.

Layer type	Description	Output shape
Input		(H, W, C)
Convolution, BN, ReLU	3×3 filter, stride S, padding 1	(H/S, W/S, OC)
Convolution, BN	3×3 filter, stride 1, padding 1	(H/S, W/S, OC)
Residual Connection	Adds the input tensor to the output tensor	(H/S, W/S, OC)
ReLU	Applies ReLU activation function	(H/S, W/S, OC)

The output shape is given as (height, width, channels). H, W, and C denote the height, width, and number of channels of the input, respectively. S denotes the stride of the convolution. OC denotes number of channels of the output.

**Table 4:** Network architecture of the LSTM model.

Layer type	Description	Output shape
Input	4700 genotypes	(batch, 47, 100)
LSTM	LSTM layer with HD units	(batch, 47, HD)
Hidden State Selection	Select the last hidden state	(batch, HD)
Dropout		(batch, HD)
Fully Connected Output		(batch, 4)

Batch denotes the batch size. HD denotes the number of hidden units.

**Table 5:** The evaluation performance of the deep learning models in predicting the four phenotypes.

Model	Top-1 Accuracy	Top-2 Accuracy	Sensitivity	Specificity	Precision	F1 Score	Micro AUC	Macro AUC
CNN	0.654	0.898	0.653	0.877	0.672	0.659	0.89	0.86
ResNet18	0.693	0.898	0.671	0.889	0.696	0.682	0.88	0.85
LSTM	0.693	0.883	0.693	0.894	0.685	0.687	0.88	0.86
Ensemble	**0.693**	0.894	0.683	0.890	0.692	**0.687**		

Top-1 Accuracy denotes the proportion of correct predictions among the total predictions. Top-2 Accuracy denotes the proportion of correct predictions where the true class is among the top two predicted classes. Sensitivity, also known as recall or true positive rate. Specificity denotes the true negative rate. Precision denotes the proportion of true positive predictions out of all positive predictions made by the model. F1 Score denotes the harmonic mean of precision and sensitivity, providing a single metric that balances the trade-off between false positives and false negatives. Micro AUC denotes the AUC value calculated globally by considering all true positives, false positives, and false negatives across all classes, representing overall model performance. Macro AUC denotes the AUC value calculated for each class independently and then averaged by treating all classes equally, and highlights performance discrepancies among different classes.

**Table 6:** The evaluation performance of the deep learning models in predicting the three phenotypes.

Model	Top-1 Accuracy	Top-2 Accuracy	Sensitivity	Specificity	Precision	F1 Score	Micro AUC	Macro AUC
CNN	0.838	0.966	0.847	0.907	0.842	0.844	0.95	0.94
ResNet18	0.846	0.979	0.861	0.910	0.861	0.861	0.95	0.94
LSTM	0.863	0.983	0.865	0.922	0.874	0.869	0.95	0.93
Ensemble	**0.863**	**0.987**	**0.873**	0.921	0.865	**0.869**		
Improved by	24.8%	9.6%	27.6%	3.1%	25.4%	26.8%	7.5%	9.3%

"Improved by" values represent the percentage increased in each metric by averaging over the four models (CNN, ResNet18, LSTM, and ensemble) when predicting three phenotypes compared to four phenotypes.

**Table 7:** The evaluation performance of models on the full-set of 4722 genotypes and the non-redundant 175-genotype set.

Model	Top-1 Accuracy	Top-2 Accuracy	Sensitivity	Specificity	Precision	F1 Score	Micro AUC	Macro AUC
CNN	0.838	0.966	0.847	0.907	0.842	0.844	0.95	0.94
ResNet18	0.846	0.979	0.861	0.910	0.861	0.861	0.95	0.94
LSTM	0.863	0.983	0.865	0.922	0.874	0.869	0.95	0.93
CNN_175	0.816	0.974	0.83	0.897	0.836	0.832	0.95	0.94
ResNet18_175	0.791	0.962	0.792	0.882	0.801	0.796	0.93	0.92
LSTM_175	0.791	0.979	0.812	0.881	0.818	0.813	0.94	0.93
LR_175	0.808	0.966	0.812	0.892	0.831	0.819	0.94	0.93
full vs. 175	6.21%	0.45%	5.71%	2.97%	4.97%	5.45%	1.06%	0.72%
AI vs. LR	5.07%	1.04%	5.62%	2.35%	3.37%	4.76%	1.06%	0.72%

LR denotes the Logistic regression model. “full vs. 175” represents the percentage improvement in the average values of each metric for the three AI models trained on the complete loci set, compared to the average values of the same metrics for the three AI models trained on the reduced 175 loci set for three-phenotype prediction. “AI vs. LR” indicates the percentage improvement in the average values of each metric for the three AI models trained on the complete loci set, compared to the values of the same metrics for the logistic regression model trained on the reduced 175 loci set for three-phenotype prediction.

## References

[1] Carapetis JR, Steer AC, Mulholland EK, Weber M (2005). The global burden of group A streptococcal diseases. Lancet Infect. Dis..

[2] Walker MJ, Barnett TC, McArthur JD, Cole JN, Gillen CM, Henningham A (2014). Disease manifestations and pathogenic mechanisms of Group A *Streptococcus*. Clin. Microbiol. Rev..

[3] Cole JN, Barnett TC, Nizet V, Walker MJ (2011). Molecular insight into invasive group A streptococcal disease. Nat. Rev. Microbiol..

[4] Power RA, Parkhill J, de Oliveira T (2017). Microbial genome-wide association studies: lessons from human GWAS. Nat. Rev. Genet..

[5] Sumby P, Whitney AR, Graviss EA, DeLeo FR, Musser JM (2006). Genome-wide analysis of group a streptococci reveals a mutation that modulates global phenotype and disease specificity. PLoS Pathog..

[6] Turner CE, Abbott J, Lamagni T, Holden MT, David S, Jones MD (2015). Emergence of a new highly successful acapsular group A *Streptococcus* clade of genotype *emm89* in the United Kingdom. mBio.

[7] Beres SB, Kachroo P, Nasser W, Olsen RJ, Zhu L, Flores AR (2016). Transcriptome remodeling contributes to epidemic disease caused by the human pathogen *Streptococcus pyogenes*. mBio.

[8] Zhu L, Olsen RJ, Nasser W, Beres SB, Vuopio J, Kristinsson KG (2015). A molecular trigger for intercontinental epidemics of group A *Streptococcus*. J. Clin. Invest..

[9] Davies MR, Holden MT, Coupland P, Chen JH, Venturini C, Barnett TC (2015). Emergence of scarlet fever *Streptococcus pyogenes* emm12 clones in Hong Kong is associated with toxin acquisition and multidrug resistance. Nat. Genet..

[10] You Y, Davies MR, Protani M, McIntyre L, Walker MJ, Zhang J (2018). Scarlet fever epidemic in China caused by *Streptococcus pyogenes* serotype M12: epidemiologic and molecular analysis. EBioMedicine.

[11] Tse H, Bao JY, Davies MR, Maamary P, Tsoi HW, Tong AH (2012). Molecular characterization of the 2011 Hong Kong scarlet fever outbreak. J. Infect. Dis..

[12] Brouwer S, Barnett TC, Ly D, Kasper KJ, De Oliveira DMP, Rivera-Hernandez T (2020). Prophage exotoxins enhance colonization fitness in epidemic scarlet fever-causing *Streptococcus pyogenes*. Nat. Commun..

[13] Kachroo P, Eraso JM, Beres SB, Olsen RJ, Zhu L, Nasser W (2019). Integrated analysis of population genomics, transcriptomics and virulence provides novel insights into *Streptococcus pyogenes* pathogenesis. Nat. Genet..

[14] Bao YJ, Shapiro BJ, Lee SW, Ploplis VA, Castellino FJ (2016). Phenotypic differentiation of *Streptococcus pyogenes* populations is induced by recombination-driven gene-specific sweeps. Sci. Rep..

[15] Davies MR, McIntyre L, Mutreja A, Lacey JA, Lees JA, Towers RJ (2019). Atlas of group A streptococcal vaccine candidates compiled using large-scale comparative genomics. Nat. Genet..

[16] Grinberg NF, Orhobor OI, King RD (2020). An evaluation of machine-learning for predicting phenotype: studies in yeast, rice, and wheat. Mach Learn..

[17] Karlsen ST, Rau MH, Sánchez BJ, Jensen K, Zeidan AA (2023). From genotype to phenotype: computational approaches for inferring microbial traits relevant to the food industry. FEMS Microbiol. Rev..

[18] Zhou L, Pan S, Wang J, Vasilakos AV (2017). Machine learning on big data: opportunities and challenges. Neurocomputing.

[19] Xu X, Liang T, Zhu J, Zheng D, Sun T (2019). Review of classical dimensionality reduction and sample selection methods for largescale data processing. Neurocomputing.

[20] Ching T, Himmelstein DS, Beaulieu-Jones BK, Kalinin AA, Do BT, Way GP (2018). Opportunities and obstacles for deep learning in biology and medicine. J. R. Soc. Interface.

[21] Mahmud M, Kaiser MS, Hussain A, Vassanelli S (2018). Applications of deep learning and reinforcement learning to biological data. IEEE Trans. Neural Netw. Learn. Syst..

[22] Sapoval N, Aghazadeh A, Nute MG, Antunes DA, Balaji A, Baraniuk R (2022). Current progress and open challenges for applying deep learning across the biosciences. Nat. Commun..

[23] Sharma D, Xu W (2021). phyLoSTM: a novel deep learning model on disease prediction from longitudinal microbiome data. Bioinformatics.

[24] Alzoubi H, Alzubi R, Ramzan N (2023). Deep Learning framework for complex disease risk prediction using genomic variations. Sensors (Basel).

[25] Schmauch B, Romagnoni A, Pronier E, Saillard C, Maillé P, Calderaro J (2020). A deep learning model to predict RNA-Seq expression of tumours from whole slide images. Nat. Commun..

[26] Ding K, Dixit G, Parker BJ, Wen J (2023). CRMnet: a deep learning model for predicting gene expression from large regulatory sequence datasets. Front. Big Data.

[27] Patiyal S, Dhall A, Raghava GPS (2022). A deep learning-based method for the prediction of DNA interacting residues in a protein. Brief. Bioinform..

[28] Li J, Chiu TP, Rohs R (2024). Predicting DNA structure using a deep learning method. Nat. Commun..

[29] Nguembang Fadja A, Riguzzi F, Bertorelle G, Trucchi E (2021). Identification of natural selection in genomic data with deep convolutional neural network. BioData Min..

[30] Isildak U, Stella A, Fumagalli M (2021). Distinguishing between recent balancing selection and incomplete sweep using deep neural networks. Mol. Ecol. Resour..

[31] Korfmann K, Gaggiotti OE, Fumagalli M (2023). Deep learning in population genetics. Genome Biol. Evol..

[32] Adrion JR, Galloway JG, Kern AD (2020). Predicting the landscape of recombination using deep learning. Mol. Biol. Evol..

[33] Flagel L, Brandvain Y, Schrider DR (2019). The unreasonable effectiveness of convolutional neural networks in population genetic inference. Mol. Biol. Evol..

[34] Fang Y, Deng S, Li C (2022). A generalizable deep learning framework for inferring fine-scale germline mutation rate maps. Nat. Mach. Intell..

[35] Nguyen ND, Huang J, Wang D (2022). A deep manifold-regularized learning model for improving phenotype prediction from multimodal data. Nat. Comput. Sci..

[36] Ma W, Qiu Z, Song J, Li J, Cheng Q, Zhai J (2018). A deep convolutional neural network approach for predicting phenotypes from genotypes. Planta.

[37] Peng-Ying Wang, Zhong Liang, Zhi-Song Chen, Yun-Juan Bao, Castellino FJ (2024). compGWAS: a new GWAS tool allows revelation of the genetic architecture and risk stratification for the versatile pathogen *Streptococcus pyogenes*.

[38] Stirling JR, Zakynthinaki M (2008). The point of maximum curvature as a marker for physiological time series. J. Nonlinear Math. Phys..

[39] He K, Zhang X, Ren S, Sun J, editors. Deep Residual Learning for Image Recognition. 2016 IEEE Conference on Computer Vision and Pattern Recognition (CVPR); 2016 27-30 June 2016.

[40] Sak H, Senior A, Beaufays F (2014). Long short-term memory recurrent neural network architectures for large scale acoustic modeling. Proceedings of the Annual Conference of the International Speech Communication Association, INTERSPEECH..

[41] Zeiler MD, Fergus R, editors. Visualizing and Understanding Convolutional Networks. Computer Vision – ECCV 2014; 2014 2014/ /; Cham: Springer International Publishing.

[42] Koushik J. Understanding convolutional neural networks. arXiv preprint arXiv:160509081. 2016.

[43] Szegedy C, Ioffe S, Vanhoucke V, Alemi A. Inception-v4, Inception-ResNet and the Impact of Residual Connections on Learning. AAAI Conference on Artificial Intelligence. 2016;31. doi: 10.1609/aaai.v31i1.11231. 10.1609/aaai.v31i1.11231

[44] Shafiq M, Gu Z (2022). Deep residual learning for image recognition: a survey. Appl. Sci..

[45] Schmidhuber J, Hochreiter S (1997). Long short-term memory. Neural Comput..

[46] Greff K, Srivastava RK, Koutník J, Steunebrink BR, Schmidhuber J (2016). LSTM: a search space odyssey. IEEE Trans. Neural Netw. Learn. Syst..

[47] Castelvecchi D (2016). Can we open the black box of AI?. Nature.

[48] Rudin C (2019). Stop Explaining black box machine learning models for high stakes decisions and use interpretable models instead. Nat. Mach. Intell..

[49] Jones Y, Deligianni F, Dalton J, editors. Improving ECG classification interpretability using saliency maps. 2020 IEEE 20th International Conference on Bioinformatics and Bioengineering (BIBE); 2020: IEEE.

[50] Ismail AA, Corrada Bravo H, Feizi S (2021). Improving deep learning interpretability by saliency guided training. Adv. Neural Inform. Processing Syst..

[51] Linardatos P, Papastefanopoulos V, Kotsiantis S (2020). Explainable AI: a review of machine learning interpretability methods. Entropy (Basel).

[52] Johnson M, Albizri A, Harfouche A, Fosso-Wamba S (2022). Integrating human knowledge into artificial intelligence for complex and ill-structured problems: informed artificial intelligence. Int. J. Inform. Manag..

[53] Holzinger A, Saranti A, Hauschild A-C, Beinecke J, Heider D, Roettger R. editors. Human-in-the-loop integration with domainknowledge graphs for explainable federated deep learning. International Cross-Domain Conference for Machine Learning and Knowledge Extraction; 2023: Springer.

[54] Manzoni C, Kia DA, Vandrovcova J, Hardy J, Wood NW, Lewis PA (2018). Genome, transcriptome and proteome: the rise of omics data and their integration in biomedical sciences. Brief. Bioinform..

[55] Biswas N, Chakrabarti S (2020). Artificial Intelligence (AI)-based systems biology approaches in multi-omics data analysis of cancer. Front. Oncol..

[56] Cembrowska-Lech D, Krzemińska A, Miller T, Nowakowska A, Adamski C, Radaczyńska M (2023). An integrated multi-omics and artificial intelligence framework for advance plant phenotyping in horticulture. Biology (Basel).

